# Mobile phone addiction and depression among adolescents: the moderation effect of family environment

**DOI:** 10.3389/fpubh.2026.1863623

**Published:** 2026-06-30

**Authors:** Yanlin Bai, Xia Xu, Weijie Jiang, Yuting Yang, Qixi Li, Xiaoli Li, Qian Zhu, Yajing Meng, Changjian Qiu

**Affiliations:** 1 Mental Health Center, National Center for Mental Disorders, West China Hospital, Sichuan University, Chengdu, China; 2 Leshan Psychosomatic Hospital, Leshan, China; 3 Department of Sociology and Psychology, School of Public Administration, Sichuan University, Chengdu, China

**Keywords:** adolescent mental health, depression, family environment typology, latent class analysis, Mobile phone addiction, moderation effect

## Abstract

**Background:**

Mobile phone addiction (MPA) is positively associated with adolescent depressive symptoms, yet the moderating role of multidimensional family environments in this relationship remains poorly understood. This study aimed to identify distinct family environment typologies and examine their moderating effects on the MPA-depression association among Chinese adolescents.

**Methods:**

A cross-sectional, school-based survey was conducted among middle and high school students in China. MPA and depressive symptoms were assessed using the Mobile Phone Addiction Index (MPAI) and the Patient Health Questionnaire-9 (PHQ-9), respectively. Latent class analysis (LCA) was employed to identify family environment typologies based on seven socioeconomic and structural indicators. Moderated regression analyses tested interaction effects, adjusting for age and gender.

**Results:**

A total of 103,874 students (mean age 15.58 ± 1.74; 50.4% female) participated. LCA revealed three family clusters: “*High-Resource Stable Family*,” “*Low-Resource Cohesive Family*,” and “*Low-Resource Fragmented Family*.” Adolescents in *Low-Resource Fragmented family* had significantly higher MPA and depression scores (*p* < 0.001). MPA was positively correlated with depression across all groups (*p* < 0.001). Family type significantly moderated this relationship, with stronger associations in both Low-Resource clusters (*p* < 0.05). Subgroup analyses showed specific moderation effects by gender and school level.

**Conclusion:**

Multidimensional family environments not only shape levels of MPA and depression but also amplify their association in socioeconomically disadvantaged and structurally fragmented contexts. Employing LCA to identify family typologies provides a nuanced, person-centered framework for targeting prevention efforts and identifying adolescents at elevated digital mental health risk.

## 1 Introduction

With the proliferation of digital technology, mobile phones have become integral to daily life. As of December 2023, China’s internet penetration rate reached 75.5%, with 1.092 billion internet users, 1.091 billion of whom access the internet via mobile phones, indicating a 99.9% mobile adoption rate among users. While smartphones support numerous functions such as communication, information access, and entertainment (1, 2), their excessive use can lead to mobile phone addiction (MPA), a growing behavioral problem characterized by loss of control and negative impacts on everyday functioning (3, 4). MPA is particularly concerning during adolescence, a critical developmental stage marked by substantial neurobiological, psychological, and social changes (5). Neurobiologically, the prefrontal cortex, which is involved in impulse control and executive functioning, is still developing during adolescence, whereas socio-affective and reward-related neural systems are highly reactive. This developmental imbalance may increase adolescents’ susceptibility to the rewarding and compulsive aspects of smartphone use (6). Psychosocially, adolescents place greater importance on peer relationships and social acceptance, making mobile phones a primary tool for maintaining social connection and increasing vulnerability to fear of missing out and maladaptive usage patterns (7, 8). In addition, adolescence is often accompanied by increased parent–child conflict and reduced family support as individuals seek greater autonomy. Together with heightened emotional sensitivity and immature self-regulation, these developmental characteristics make adolescents especially vulnerable to the negative mental health effects of problematic digital behaviors (9). A growing body of evidence has demonstrated that MPA is associated with a range of adverse outcomes, including physical health problems, poor academic performance, anxiety, and depressive symptoms (10, 11). Given the widespread use of smartphones among adolescents and the potential impact of MPA on mental health, it is important to further investigate the mechanisms and contextual factors underlying the relationship between MPA and depression.

MPA and depressive symptoms in adolescents is well-documented in the literature (11). However, beyond merely establishing this correlation, it is crucial to elucidate the potential psychological and social mechanisms. Research suggests that one primary pathway is the displacement of face-to-face social interactions (10, 12). Excessive usage of mobile phone can diminish in-person social connection, thereby reducing the quality and quantity of social support received, which can exacerbate feelings of loneliness and isolation, ultimately contributing to depression (13). Furthermore, smartphones often serve as a tool for experiential avoidance, where adolescents use them to escape or alleviate negative emotions stemming from academic or familial pressures (14). While providing short-term relief, this avoidance coping strategy can paradoxically reinforce emotional dysregulation and prevent the development of adaptive coping skills, creating a self-perpetuating cycle that worsens both MPA and depressive symptoms over time (15). Importantly, the relationship between MPA and depressive symptoms is widely considered to be bidirectional (16). On the one hand, excessive smartphone use may intensify depressive symptoms by displacing offline social interactions. On the other hand, adolescents with depressive symptoms may increasingly rely on smartphones to avoid negative emotions, seek social reassurance, or alleviate loneliness, thereby reinforcing problematic smartphone use and creating a vicious cycle (17). Nevertheless, longitudinal evidence suggests that media use behaviors may more strongly predict subsequent depressive symptoms in adolescents than the reverse pathway (18, 19). Accordingly, the present cross-sectional study adopts a theoretically driven model in which MPA is examined as a predictor of depressive symptoms. Critically, the strength of these pathways, the degree to which social support is diminished or avoidance is reinforced is unlikely to be uniform across all adolescents. Instead, their impact is contingent upon the individual’s broader environmental context, pointing to the necessity of identifying key contextual moderators. The family environment, as the primary and most proximal social context for most adolescents, emerges as a paramount candidate for understanding these differential vulnerabilities.

The family environment is widely recognized as a cornerstone in shaping adolescent development, influencing both behavioral tendencies like MPA and mental health outcomes. Existing research has delineated the impact of several discrete family dimensions. From an economic perspective, low family socioeconomic status is associated with limited access to alternative leisure activities, which may increase vulnerability to MPA as a readily available outlet and, concurrently, elevate the risk for depression (20, 21). From a structural perspective, factors such as being a non-only child or experiencing parental separation can correlate with reduced parental supervision and increased family conflict, creating an emotional climate that fosters both problematic phone use and psychological distress (22). Furthermore, aspects of family functioning and relationships, including low family cohesion and ineffective parental monitoring, have been independently linked to higher levels of adolescent MPA and internalizing symptoms (23). Although these findings highlight the importance of family factors, most existing studies have adopted a variable-centered approach that examines the independent effects of isolated family characteristics. Such approaches assume population homogeneity and fail to capture how multiple family risks and protective factors interact and co-occur within real-world family contexts. Consequently, the broader configurations or typologies of family environments, and their associations with adolescent well-being, remain poorly understood. To address this limitation, a person-centered approach such as Latent Class Analysis (LCA) is warranted. Unlike traditional variable-centered methods, LCA identifies latent subgroups of individuals who share similar patterns across multiple indicators. This approach enables the characterization of holistic family environment profiles that more accurately reflect the complexity of adolescents’ lived experiences.

To establish a conceptual framework, we examine how chronic stressors and protective resources within the family system interact to shape adolescent vulnerability. Socioeconomic disadvantages can trigger multiple stressors, such as parental distress and family conflict, which increase the risk for both maladaptive coping behaviors like MPA and the onset of depressive symptoms. Conversely, a cohesive and stable family environment can buffer against these stressors. We thus propose that families experiencing combined economic strain and structural instability represent a context of heightened risk with minimal protection. In these high-risk contexts, the association between MPA and depression is expected to be most pronounced. Using Latent Class Analysis (LCA) to identify holistic family profiles, we test the following hypotheses:

*Hypothesis 1 (H1)*: Latent Class Analysis will identify distinct and meaningful typologies of family environment based on socioeconomic, structural, and functional characteristics. Adolescents in family typologies characterized by higher levels of socioeconomic adversity and instability will report significantly higher levels of both mobile phone addiction and depressive symptoms compared to those in typologies characterized by greater resources and stability.

*Hypothesis 2 (H2)*: The identified family environment typology will significantly moderate the positive association between mobile phone addiction and depressive symptoms. Specifically, this positive association will be strongest in the most adverse and vulnerable family typologies and weakest in the most resourceful and stable ones.

## 2 Method

### 2.1 Study design and participants

This cross-sectional study utilized data from an annual school-based mental health survey conducted in September 2023 among middle and high school students in Leshan, Sichuan Province, China, with approval from the Medical Ethics Committee of Leshan Psychiatric Hospital ([2024] 03). The sampling consisted of general junior high and senior high schools in Leshan. Vocational high schools were excluded from the sampling frame due to their distinct educational structure, curriculum focus, and student demographic profiles, which may be associated with different patterns of mobile phone use and family dynamics. This decision was made to enhance sample homogeneity and ensure the comparability of findings within the general middle and high school population.

This study was conducted as part of a large-scale, school-based mental health census commissioned by local health and education authorities to generate regionally representative data on adolescent well-being. The population-based design minimized sampling bias and enhanced the generalizability of the findings to adolescents in Leshan, thereby providing a robust evidence base for policy development.

### 2.2 Procedure

The survey was administered in school computer rooms via the “Online School Student Mental Health Management System,” designed for provincial school-based mental health surveys. The questionnaire included demographic items and psychological measures, taking approximately 8 min to complete. Teachers trained by the research team provided clarifications without influencing responses. Informed consent was obtained from participants and guardians electronically. Data were collected via an electronic questionnaire beginning with demographic details (including student ID for tracking), followed by psychological instruments. Teachers provided standardized clarification on items when needed.

### 2.3 Outcomes

Baseline characteristics included: grade, boarding status, only-child status, current living situation, cohabiting relatives, experience of separation from parents for >6 months due to migrant work, household composition, parental occupation and education, family financial status, annual household income, satisfaction with family relationships, frequency of miscommunication, peer conflicts, personal or family severe medical history, bereavement in the past 12 months, and chronic illness or long-term medication use. Seven items (only-child status, current living conditions, cohabitation with relatives, history of parental separation (>6 months), perceived family financial status, annual household income, and satisfaction with family relationships) were used in latent class analysis as family environment indicators.

#### 2.3.1 The 17-item mobile phone addiction index (MPAI)

Mobile phone addiction was assessed using the 17-item Mobile Phone Addiction Index (MPAI), originally developed by Louis Leung (24) and cross-culturally adapted for Chinese populations by Huang et al. (25). The scale employs a 5-point Likert-type scoring system (1 = “not at all” to 5 = “always”), with total scores ranging from 17 to 85, where higher scores indicate greater addiction severity. The scale includes four dimensions: inability to control craving, anxiety and feeling lost, productivity loss, and withdrawal and escape. In the current sample, the MPAI demonstrated good internal consistency, with a Cronbach’s *α* of 0.923 for the total scale. The four subscales also showed acceptable to good reliability: inability to control craving (α = 0.862), anxiety and feeling lost (α = 0.803), productivity loss (α = 0.821), and withdrawal and escape (α = 0.801).

#### 2.3.2 The 9-item patient health questionnaire (PHQ-9)

Depressive symptoms were evaluated using the 9-item Patient Health Questionnaire (PHQ-9), a validated screening tool designed to assess symptom severity over the preceding two-week period (26). The PHQ-9 comprises nine items measuring core depressive manifestations: anhedonia, dysphoric mood, sleep disturbances, fatigue, appetite changes, self-critical thoughts, concentration difficulties, psychomotor agitation/retardation, and suicidal ideation. Each item is rated on a 4-point Likert-type scale (0 = “not at all,” 1 = “several days,” 2 = “more than half the days,” 3 = “nearly every day”), yielding a total score ranging from 0 to 27. Consistent with established clinical thresholds, a cutoff score of ≥10 was employed to identify participants with clinically significant depressive symptoms (sensitivity: 88%; specificity: 88%). Symptom severity was further categorized as asymptomatic (0–4), mild (5–9), moderate (10–14), moderately severe (15–19), and severe (≥20) (27). The PHQ-9 has demonstrated robust psychometric properties for detecting major depression and evaluating suicide risk, with summed item scores providing a composite measure of both affective-cognitive and somatic symptom domain. In the present sample, the PHQ-9 yielded a Cronbach’s *α* of 0.647.

### 2.4 Statistical analysis

#### 2.4.1 Latent class analysis (LCA)

Latent class analysis (LCA) was conducted using the *poLCA* package in R (version 4.1.1) (28) to identify distinct subtypes based on seven categorical family-related indicators: only-child status, current living conditions, cohabitation with relatives, history of parental separation (>6 months), perceived family financial status, annual household income, and satisfaction with family relationships. Model parameters were estimated via maximum likelihood, with posterior probabilities of class membership being computed for each participant (29). Model selection was guided by consistent Akaike information criterion (CAIC), sample-size adjusted Bayesian information criterion (SABIC), likelihood ratio/deviance statistics, and entropy. Lower values of CAIC and SABIC, coupled with higher entropy and average latent class posterior probabilities, were used to determine the optimal model. After evaluating models with 2–6 classes, the 3-class solution demonstrated superior statistical fit and clinical interpretability (Supplementary Table S1). Radar charts were generated to visualize class-specific response patterns by plotting normalized deviations from the overall sample proportions for each indicator.

#### 2.4.2 Comparison of group differences across the identified three subtypes and correlation analysis

For continuous variables, one-way ANOVA with Bonferroni post-hoc tests was used to compare the three latent classes. Ordinal variables were analyzed using the Kruskal–Wallis test followed by Dunn’s test, and nominal variables were assessed with chi-square tests with Bonferroni-adjusted pairwise comparisons. Pearson correlation analysis was conducted to examine associations between PHQ-9 and MPAI total scores in the overall sample and within each latent class.

#### 2.4.3 Moderation analysis

To investigate the moderating role of family structure in the relationship between mobile phone addiction and depressive symptom, we conducted moderated regression analyses. Mobile phone dependency (MPAI total score) was entered as the independent variable and depressive symptom severity (PHQ-9 total score) as the dependent variable. Family-related latent classes were included as a nominal (polytomous) moderator, dummy variables were created for each non-reference class and latent class 1 served as the reference category. Age and gender were included as covariates and entered into the model as control variables. The regression was performed in three steps: (1) covariates (age, gender); (2) main effects (MPAI and class dummies); and (3) interaction terms between MPAI and each class dummy (MPAI × class_k). To examine the moderating role of family structure across different genders and school levels, we conducted a subgroup analysis. The Bonferroni correction was applied to adjust for multiple testing.

All analyses were performed in R (version 4.1.1). Two-tailed *p*-values <0.05 were considered statistically significant.

## 3 Results

A total of 104,028 students from these participating schools were invited to complete the survey, of whom 103,874 students (response rate: 99.9%) successfully submitted valid questionnaires. Mandatory response settings in the electronic survey ensured that no item-level missing data occurred. Exclusion criteria included absence on the assessment day, difficulty understanding questionnaire items despite assistance, or incomplete submission. The final analytic sample comprised 103,874 students (50.4% female), with a mean age of 15.58 years (SD = 1.74; range 11 to 21). Clinical assessment results revealed the following mean scores across measurement scales: the MPAI yielded a total score of 31.49 (SD = 12.50), PHQ-9 demonstrated a depression severity score of 6.36 (SD = 5.30).

### 3.1 Latent class analysis of family characteristics

To determine the optimal number of latent classes, models comprising two to six classes were compared using CAIC, SABIC, deviance, entropy, and average posterior probabilities (Supplementary Table S1). Although model fit indices generally improved as the number of classes increased, the three-class solution was selected based on parsimony and substantive interpretability. The two-class solution was overly simplistic, as it combined the distinct “Low-Resource Cohesive Family” and “Low-Resource Fragmented Family” profiles into a single heterogeneous class, thereby obscuring important differences in family structure, perceived support, and mental health outcomes. In contrast, the four-class solution yielded an additional class that was highly similar to the High-Resource Stable Family class in terms of socioeconomic and structural characteristics, suggesting unnecessary fragmentation of an existing profile without providing meaningful theoretical or clinical insight. The five- and six-class solutions showed lower classification quality, with declining average posterior probabilities (AvePP < 0.76), and generated several small and conceptually redundant classes. Among all candidate models, the three-class solution demonstrated the highest entropy (0.651), indicating the greatest degree of class separation and the most reliable classification of individuals into distinct family typologies. Also, the class-specific average posterior probabilities for classes 1, 2, and 3 were 0.8731, 0.8461 and 0.8076 (Supplementary Table S2), respectively, showing excellent classification certainty. Therefore, the three-class solution was retained for all subsequent group comparisons and moderation analyses.

**Figure 1:**
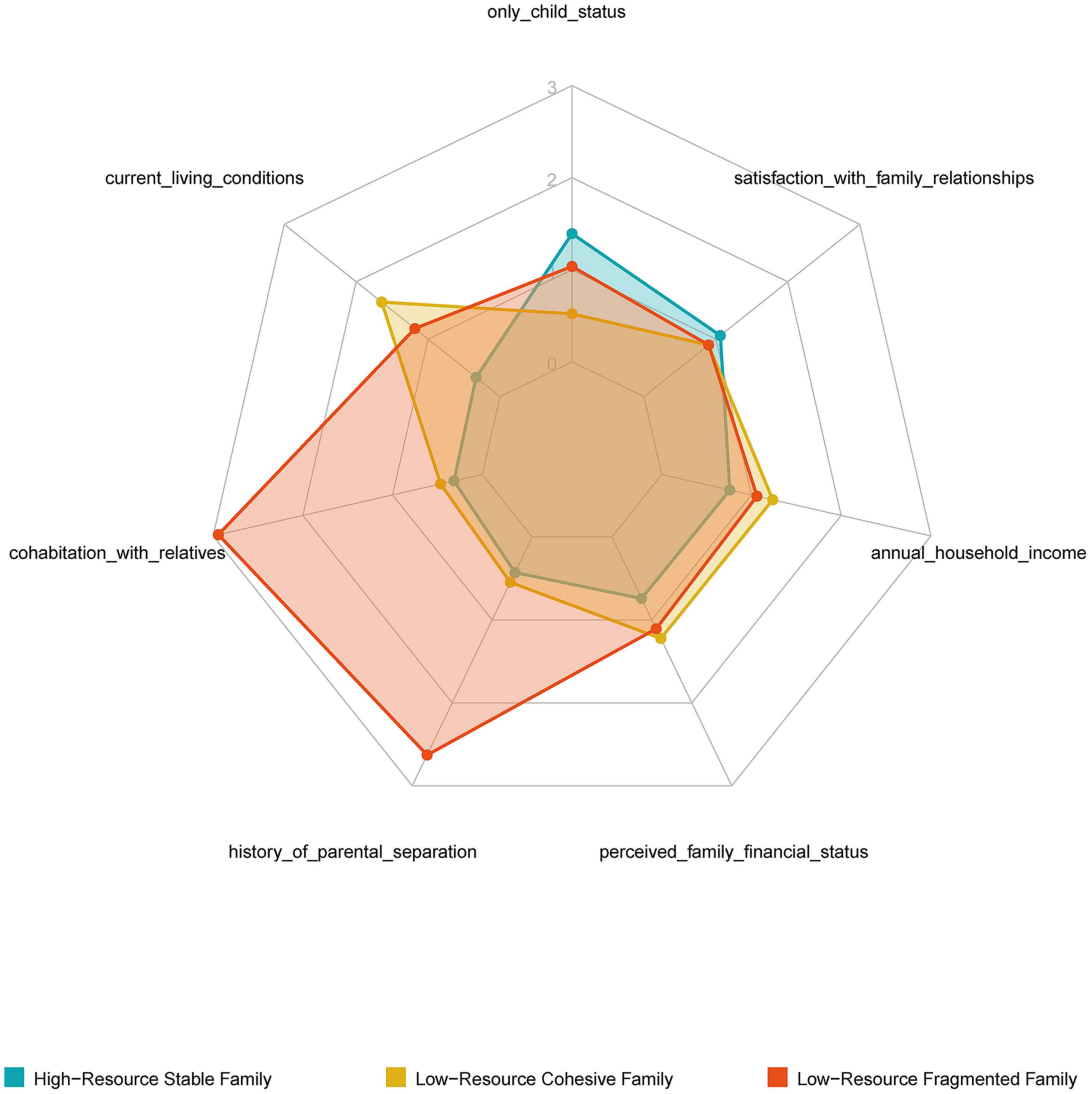
The identified family-associated subtypes. The y-values (ranging from 0 to 3) for each family-related variable represent the relative level within identified cluster compared to the total population, with the following specific interpretations:binary variables are coded as 0 (non-only child) and 1 (only child) for only child status, 0 (no parental separation >6 months) and 1 (experienced separation) for prolonged separation history, while ordinal variables (higher scores indicate poorer status) include family relationship satisfaction, gross annual household income, perceived financial situation, cohabiting relatives density, and living conditions, where elevated scores systematically reflect less favorable conditions across these dimensions.

The latent class analysis (LCA) identified three distinct family typologies based on seven family-related indicators as family structures (*N* = 103,874): Latent class 1 was labeled as “*High-Resource Stable Family*” (*n* = 42,255, 40.7%), this predominant class exhibited the most stable family configuration, with 89.0% of parents maintaining continuous cohabitation and only 15.9% reporting separation for more than 6 months. Residing predominantly in urban areas (78.3%), these families were characterized by mid-range household income levels and demonstrated the highest mean scores on family relationship satisfaction measures. Latent class 2 was labeled as “*Low-Resource Cohesive Family*” (*n* = 36,627, 35.2%), comprising predominantly rural residents, these families maintained traditional multigenerational cohabitation patterns despite economic constraints. A high proportion of them reported no prolonged parental separation, and they had moderately high family satisfaction. Latent class 3 was labeled as “*Low-Resource Fragmented Family*” (*n* = 24,992, 24.1%), they had the highest parental migration rates and this socioeconomic vulnerable group resided primarily in rural or township areas with the low annual incomes. Characterized by fragmented family structures, they reported significantly reduced family relationship satisfaction compared to other classes. The character of these latent classes can be seen in Table 1 and Figure 1.

**Table 1:** Characteristics of study participants stratified by family structure.

Characteristic	Level	Overall	Latent class 1	Latent class 2	Latent class 3		
		*n* = 103,874	*n* = 42,255	*n* = 36,627	*n* = 24,992	*t*/*χ*2	*p*
Gender (%)	F	52,326 (50.4)	20,142 (47.7)	19,196 (52.4)	12,988 (52.0)	209.92	<0.001
	M	51,548 (49.6)	22,113 (52.3)	17,431 (47.6)	12,004 (48.0)		
Age (Mean [SD])		15.58 (1.74)	15.38 (1.69)	15.72 (1.78)	15.73 (1.72)	505.92	<0.001
PHQ-9 total score (Mean [SD])		6.36 (5.30)	5.65 (4.94)	6.07 (5.22)	7.97 (5.67)	1626.85	<0.001
MPAI total score (Mean [SD])		31.49 (12.50)	30.43 (11.98)	30.51 (12.01)	34.73 (13.49)	1129.61	<0.001
Inability to control craving (Mean [SD])		13.71 (5.78)	13.22 (5.56)	13.32 (5.58)	15.12 (6.17)	1008.12	<0.001
Withdrawal and escape (Mean [SD])		6.26 (3.14)	6.04 (2.97)	6.06 (2.97)	6.90 (3.53)	712.42	<0.001
Anxiety and feeling lost (Mean [SD])		6.38 (3.16)	6.24 (3.11)	6.13 (3.02)	6.98 (3.35)	614.37	<0.001
Productivity loss (Mean [SD])		5.15 (2.65)	4.93 (2.52)	5.01 (2.54)	5.73 (2.93)	802.79	<0.001
Only-child status (%)	No	66,365 (63.9)	21,010 (49.7)	29,718 (81.1)	15,637 (62.6)	8417.8	<0.001
	Yes	37,509 (36.1)	21,245 (50.3)	6,909 (18.9)	9,355 (37.4)		
Current living conditions (%)	City	48,241 (46.4)	33,071 (78.3)	6,307 (17.2)	8,863 (35.5)	33,346	<0.001
	Country	29,895 (28.8)	2,397 (5.7)	19,266 (52.6)	8,232 (32.9)		
	Villages	25,738 (24.8)	6,787 (16.1)	11,054 (30.2)	7,897 (31.6)		
Cohabitation with relatives (%)	Both parents work outside the home (more than 6 months) and live with other relatives	9,787 (9.4)	858 (2.0)	1,034 (2.8)	7,895 (31.6)	54,063	<0.001
	Live with parents	71,135 (68.5)	37,612 (89.0)	31,286 (85.4)	2,237 (9.0)		
	Living in a social welfare institution or other	964 (0.9)	106 (0.3)	456 (1.2)	402 (1.6)		
	One of the parents works outside the home (more than 6 months) and lives with the other parent	21,988 (21.2)	3,679 (8.7)	3,851 (10.5)	14,458 (57.9)		
History of parental separation (>6 months) (%)	no	65,482 (63.0)	35,546 (84.1)	29,223 (79.8)	713 (2.9)		
	Yes	38,392 (37.0)	6,709 (15.9)	7,404 (20.2)	24,279 (97.1)		
Perceived family financial status (%)	General	54,410 (52.4)	13,993 (33.1)	25,079 (68.5)	15,338 (61.4)	34,315	<0.001
	Medium	35,257 (33.9)	26,764 (63.3)	3,076 (8.4)	5,417 (21.7)		
	Poor	12,731 (12.3)	102 (0.2)	8,472 (23.1)	4,157 (16.6)		
	Rich	1,476 (1.4)	1,396 (3.3)	0 (0.0)	80 (0.3)		
Annual household income (%)	150,000–300,000	11,992 (11.5)	10,289 (24.3)	0 (0.0)	1703 (6.8)	41,420	<0.001
	60,000–150,000	40,700 (39.2)	23,669 (56.0)	6,790 (18.5)	10,241 (41.0)		
	less than 60,000	47,694 (45.9)	5,130 (12.1)	29,832 (81.4)	12,732 (50.9)		
	More than 300,000	3,488 (3.4)	3,167 (7.5)	5 (0.0)	316 (1.3)		
Satisfaction with family relationships (%)	Dissatisfied	2,814 (2.7)	652 (1.5)	694 (1.9)	1,468 (5.9)	7152.6	<0.001
	Medium	17,161 (16.5)	4,557 (10.8)	5,691 (15.5)	6,913 (27.7)		
	Satisfied	32,242 (31.0)	12,697 (30.0)	10,645 (29.1)	8,900 (35.6)		
	Very dissatisfied	1,015 (1.0)	219 (0.5)	235 (0.6)	561 (2.2)		
	Very satisfied	50,642 (48.8)	24,130 (57.1)	19,362 (52.9)	7,150 (28.6)		

Latent class 1, Participants in High-Resource Stable Family, latent class 2, Participants in Low-Resource Cohesive Family, latent class 3, Participants in Low-Resource Fragmented Family.

*p*-value: Comparison among three groups.

### 3.2 Descriptive statistics and class comparisons

The 17-item MPAI demonstrated significant between-class differences, with latent class 1 showing the lowest total scores (*M* = 30.43, SD = 11.98), followed by latent class 2 (*M* = 30.51, SD = 12.01), and latent class 3 exhibiting the highest scores (*M* = 34.73, SD = 13.49; *p* < 0.001). Similar trend was seen in the dimension of MPAI including inability to control craving, productivity loss. Specially, as for withdrawal and escape, there is no significant difference between latent class 1 and 2, and as for anxiety and feeling lost, latent class 1 showed higher score than latent class 2. A parallel pattern was observed in depression severity measured by the PHQ-9: with latent class 1 showing the lowest total scores (*M* = 5.65, SD = 4.94), followed by latent class 2 (*M* = 6.07, SD = 5.22), and latent class 3 exhibiting the highest scores (*M* = 7.97, SD = 5.67; *p* < 0.001). The result of pairwise comparisons between the three latent classes have shown in Supplementary Table S3. Gender-based analyses revealed significant disparities in mobile phone addiction patterns and depression severity, with female participants exhibiting significantly higher scores than their male counterparts in three latent classes (*p* < 0.001) (Supplementary Table S4).

### 3.3 Correlation of mobile phone addiction, depression, and family associated latent class

As for the association between family associated classes and mobile phone addiction or depression severity, we have conducted ANOVA test and post-hoc analysis, which showed that the latent classes were significantly associated with both mobile phone addiction and depression severity. And the correlation of mobile phone addiction and depression severity revealed that there was a significant correlation both in all participants and in different latent classes (Table 2). These results justified further analysis of the moderating analysis.

**Table 2:** Correlations for the mobile phone addiction and depression.

Group	Pearson’s *r*	LCI	UCI	*p*
Total	0.619	0.615	0.623	< 0.001
Latent Class 1	0.602	0.596	0.609	< 0.001
Latent Class 2	0.622	0.616	0.628	< 0.001
Latent Class 3	0.605	0.597	0.613	< 0.001

Latent Class 1, 2, and 3 same with Table 1.

#### 3.3.1 Moderating effects of family latent classes

To examine the interplay between mobile phone addiction (MPA), family-associated latent classes, and depression, family-associated latent class membership was included as a moderator in the regression models. The interaction between MPA and latent class membership was statistically significant, indicating that the association between MPA and depression varied across family-associated latent classes.

In the total sample, with Latent Class 1 (High-Resource Stable Family) serving as the reference group, significant positive interaction effects were observed for both Latent Class 2 and Latent Class 3. Specifically, adolescents in these classes exhibited a stronger positive association between MPA and depression severity than those in Latent Class 1 (Table 3). Although the interaction terms (MPA × Class 2 and MPA × Class 3) were statistically significant, they accounted for only a small proportion of additional variance in depression (Δ*R*^2^ = 0.0005, *F* = 9,898, *p* < 0.001). The standardized interaction coefficients were modest (*β* = 0.055 and β = 0.020, respectively).

**Table 3:** The moderation effect analysis.

Sample	Predictor	Coef	Std_Coef	SE	*t*	*p*	LLCI	ULCI	adjusted *R*^2^	Δ*R*^2^	*F*	*p*
Total	MPA	0.239	0.564	0.002	141.197	<0.001	0.236	0.242	0.4001	0.0005	9,898	<0.001
	Latent Class 2 (w2)	−0.407	0.062	0.08	−5.071	<0.001	−0.564	−0.25				
	Latent Class 3 (w3)	0.897	0.220	0.09	9.928	<0.001	0.72	1.074				
	Age	0.156	0.051	0.008	20.669	<0.001	0.141	0.17				
	Gender	0.862	0.081	0.026	33.552	<0.001	0.811	0.912				
	MPA × w2	0.023	0.055	0.002	9.577	<0.001	0.019	0.028				
	MPA × w3	0.009	0.020	0.003	3.345	<0.001	0.004	0.014				

Latent_Class 1, 2, and 3 same with Table 1. Latent_Class 2, 3 compared with Latent_Class 1.

Coef, Coefficient; Std_Coef, Standardized Coefficient.

**Figure 2:**
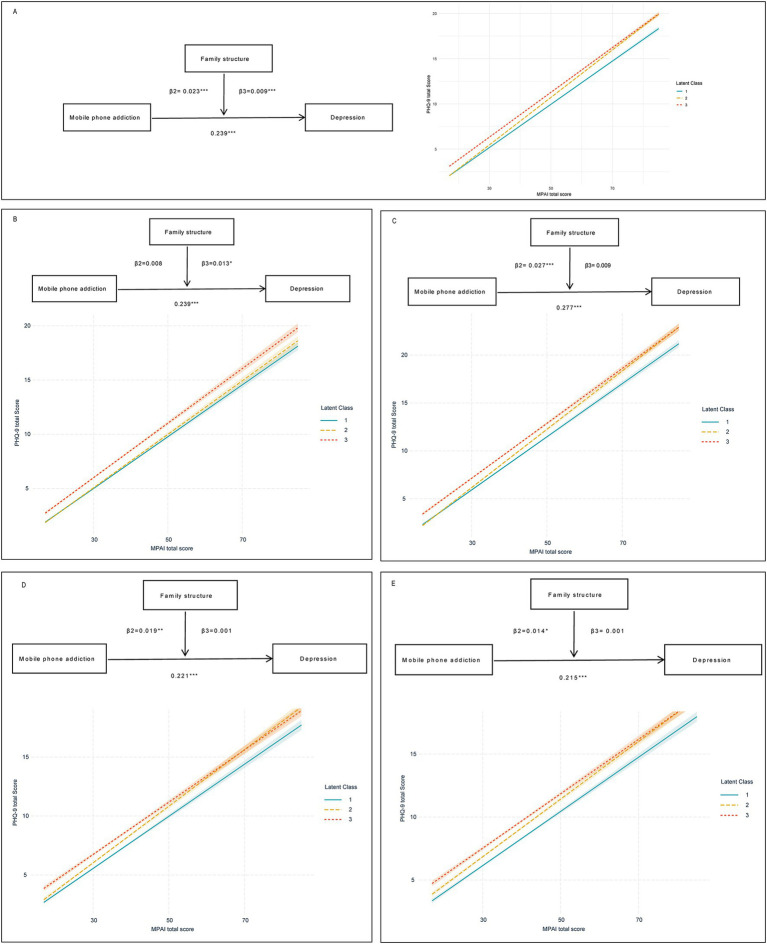
The final model of moderation analysis. **(A)** total participants, **(B)** junior high school males, **(C)** junior high school females, **(D)** senior high school males, **(E)** senior high school females.

Subgroup analyses further revealed heterogeneity in these moderation effects (Table 4 and Figure 2). For Latent Class 2, significant positive interaction effects were observed among junior high school females, senior high school males, and senior high school females. In contrast, for Latent Class 3, a significant positive interaction effect was detected only among junior high school males. Across all subgroups, the interaction terms explained a limited amount of additional variance in depression, with Δ*R*^2^ values ranging from 0.0002 to 0.0007 and standardized interaction coefficients ranging from 0.001 to 0.061.

**Table 4:** The exploration of subgroups of moderation effect analysis.

Subgroup	Predictor	Coef	Std_Coefficient	SE	*t*	*p*	P adjusted	LLCI	ULCI	adjusted *R*^2^	Δ*R*^2^	*F*	*p*
Junior male	MPA	0.239	0.564	0.003	76.523	<0.001		0.232	0.245	0.356	0.0002	3,413	<0.001
	Latent Class 2(w2)	−0.167	0.011	0.137	−1.226	0.22		−0.435	0.1				
	Latent Class 3(w3)	0.618	0.203	0.153	4.027	<0.001		0.317	0.918				
	MPA × w2	0.008	0.018	0.005	1.669	0.095	0.127	−0.001	0.017				
	MPA × w3	0.013	0.030	0.005	2.634	0.008	0.022	0.003	0.022				
Junior female	MPA	0.277	0.628	0.003	88.43	<0.001		0.271	0.284	0.453	0.0007	5,020	<0.001
	Latent Class 2(w2)	−0.564	0.045	0.143	−3.946	<0.001		−0.844	−0.284				
	Latent Class 3(w3)	0.942	0.215	0.164	5.733	<0.001		0.62	1.263				
	MPA × w2	0.027	0.061	0.005	5.969	<0.001	<0.001	0.018	0.036				
	MPA × w3	0.009	0.021	0.005	1.948	0.051	0.082	0	0.019				
Senior male	MPA	0.221	0.524	0.004	56.073	<0.001		0.213	0.229	0.309	0.0005	1854	<0.001
	Latent Class 2(w2)	−0.094	0.107	0.2	−0.469	0.639		−0.485	0.298				
	Latent Class 3(w3)	1.172	0.238	0.219	5.351	<0.001		0.743	1.601				
	MPA × w2	0.019	0.045	0.006	3.258	0.001	0.004	0.008	0.03				
	MPA × w3	0.001	0.001	0.006	0.09	0.928	0.928	−0.011	0.012				
Senior female	MPA	0.215	0.527	0.004	55.63	<0.001		0.207	0.222	0.31	0.0002	1982	<0.001
	Latent Class 2(w2)	0.289	0.155	0.207	1.398	0.162		−0.116	0.695				
	Latent Class 3(w3)	1.358	0.273	0.224	6.065	<0.001		0.919	1.797				
	MPA × w2	0.014	0.034	0.006	2.492	0.013	0.025	0.003	0.025				
	MPA × w3	0.001	0.002	0.006	0.155	0.877	0.928	−0.01	0.012				

Simple slope analyses demonstrated that the association between MPA and depression was positive and statistically significant across all latent classes and subgroups (all *p* < 0.001; Supplementary Table S5). Furthermore, predicted outcome comparisons indicated that adolescents classified as Latent Class 3 (Low-Resource Fragmented Family) consistently reported higher levels of depression than those classified as Latent Class 1 (High-Resource Stable Family). The estimated mean differences ranged from 0.97 to 1.39 points across subgroups (all *p* < 0.001; Supplementary Table S6).

## 4 Discussion

This large-scale study employed latent class analysis (LCA) to examine how multidimensional family environments moderate the association between MPA and depressive symptoms among 103,874 Chinese adolescents. We identified three distinct family typologies, among which adolescents from the most disadvantaged class, characterized by economic strain and structural instability reported the highest levels of both MPA and depressive symptoms. A consistent positive relationship between MPA and depression was observed across all groups, yet this association was significantly moderated by family class. In the total sample, the link between MPA and depression was strongest within the most resource-deprived and unstable family context. However, as the exploratory subgroup analyses revealed heterogeneity in this moderating pattern, this finding should be interpreted with caution and does not uniformly generalize across all gender and school-level groups. Our findings underscore that the family environment functions not merely as a source of risk, but as a key contextual amplifier of digital health risks during adolescence.

Our application of LCA provides methodological and conceptual advancement beyond prior research that has typically relied on isolated family variables. While it is well-established that socioeconomic disadvantages and non-intact family structures are general risk factors for adolescent psychopathology (30–32), LCA reveals how these factors co-occur to form distinct risk and protective profiles. Notably, we identified a subgroup where family stability and cohesion appeared to buffer against economic disadvantage, a protective pattern that isolated examination of single variables would likely fail to capture. Also, the study demonstrates the ability of LCA to uncover meaningful heterogeneity, such as the configuration of compounded vulnerability when economic strain coincides with parental separation and low support. By classifying the family environment as a holistic entity, our study moves beyond treating it as a background covariate and instead frames it as a central, configural context for understanding digital mental health risks.

Consistent with prior research, our findings revealed clear differences in MPA and depressive symptoms across the identified family classes. Those in the most vulnerable class, characterized by concurrent economic strain, parental separation, and weaker support systems, reported the highest scores on both outcomes. This pattern aligns with the family stress model, which posits that economic pressure can increase parental distress and conflict, thereby fostering an aversive home environment that elevates adolescents’ risk for both maladaptive coping behaviors like MPA and the development of depressive symptoms (33, 34). Conversely, the observed gradient in risk across classes underscores the multidimensional family resources, not only economic capital but also structural stability and relational support collectively shape adolescent mental health. Our findings are thus consistent with existing evidence indicating that the co-occurrence of economic and emotional resource deprivation carries cumulative risk for adverse outcomes (31, 35, 36), while also highlighting that stability in one domain may offer some protection against challenges in another.

Critically, our analysis establishes that family typology significantly moderates the MPA-depression link. In the total sample, the link between MPA and depression was strongest within the most resource-deprived and unstable family context. This moderating effect aligns with a stress-resource framework. In high-resource, stable families, strong relational support can buffer the psychological impact of MPA, likely by providing alternative coping avenues and reducing the reliance on phones for emotional regulation. Conversely, in low-resource, vulnerable families, pre-existing stressors and scant protective resources may amplify the detrimental effect of MPA, making it a potent final straw that exacerbates depressive states. This finding shifts the conceptualization of family environment from a simple source of risk to a pivotal contextual amplifier in the developmental pathway from behavioral addiction to mental illness, underscoring that the same level of MPA can carry vastly different mental health implications depending on the adolescent’s overarching family context. However, when examining the moderating effects, the observed interaction coefficients were relatively small. To further assess practical significance, we examined standardized coefficients and incremental *R*^2^. The standardized interaction coefficients ranged from 0.001 to 0.061 across models, and the Δ*R*^2^ values were consistently below 0.001. These small effect sizes suggest that although family typology statistically moderates the MPA-depression relationship, the magnitude of this moderation is limited. This may be because a strong association between mobile phone addiction and depression already exists even among adolescents from families with higher economic status and greater stability. Thus, the additional increase in the interaction coefficient attributable to declines in family economic condition and stability is limited. Furthermore, adolescents from different family structures may use mobile phones for different purposes. The relationship between mobile phone use and emotional problems could be influenced by these specific usage patterns, which warrants further investigation in future studies.

Our exploratory analyses by gender and school level revealed some variation in the moderating patterns, although these findings must be interpreted with caution. Notably, the strength of the interaction between family typology and MPA on depression was not uniform across all subgroups. Future research incorporating direct measures of parent–child interactions, phone usage motivations, and developmental nuances is needed to robustly test and explain these potential differential pathways.

These findings have important implications for both research and practice. For researchers, this study highlights the value of LCA as a person-centered approach for capturing heterogeneity in family contexts within digital mental health research. For practitioners, the findings suggest that assessment and intervention should extend beyond adolescents’ mobile phone use to include multidimensional family factors. Interventions should be tailored to family typologies. For adolescents from low-resource fragmented families, efforts should focus on strengthening family functioning and increasing social support, whereas family cohesion in low-resource cohesive families may serve as a protective factor in digital literacy and mental health interventions. For policymakers and schools, the findings indicate that universal restrictions on mobile phone use may be insufficient. Instead, family-context–sensitive screening and intervention strategies may better identify and support high-risk adolescents.

## 5 Limitations

Several limitations of this study should be considered. First, the cross-sectional design prevents any inference of causality among the examined variables. Although our theoretical framework conceptualized MPA as a predictor of depressive symptoms, reverse or bidirectional relationships are also possible. Future longitudinal studies employing cross-lagged panel designs are needed to clarify the temporal relationship between MPA and depression and to determine whether family typology moderates both directions of this association or predominantly one pathway. Second, the generalizability of our findings may be limited as the data were drawn exclusively from adolescents in Leshan City, whose specific socioeconomic context may not fully represent the diverse circumstances across China. Third, key measures, including family annual income, were self-reported by adolescents, which may introduce measurement error. However, it is noteworthy that adolescents’ perceived family socioeconomic status has been demonstrated as a robust predictor of their mental health outcomes. Fourth, depression was assessed using the self-report PHQ-9 rather than a structured clinical interview, which may have introduced some misclassification. The internal consistency in the present sample was modest (Cronbach’s *α* = 0.647). This likely reflects range restriction, as large community-based adolescent samples typically report predominantly low to mild symptom levels, thereby reducing variability. Such restriction can systematically attenuate reliability estimates without indicating poor scale properties. Thus, the modest α may be partly attributable to sample homogeneity, and the observed MPA–depression associations may be conservative. Future studies should adopt depression measures with stronger psychometric properties, apply more advanced psychometric approaches to model symptom heterogeneity, or include clinical samples with wider symptom spectra to further validate these findings. Fifth, some conceptual overlap may exist between certain dimensions of the MPAI and depressive symptoms assessed by the PHQ-9, particularly regarding emotional distress and functional impairment. This overlap may have partially inflated the observed association between mobile phone addiction and depression. Future studies should employ more differentiated measures and multi-method assessments to better distinguish problematic mobile phone use from depressive symptomatology. Finally, a further limitation concerns the inherent classification uncertainty associated with LCA. Although a parsimonious and clinically interpretable three-class solution was identified, subsequent between-class comparisons and moderation analyses were conducted using participants’ most-likely class assignments rather than incorporating posterior classification probabilities. Consequently, potential misclassification cannot be entirely excluded and may have attenuated or biased the estimated group differences and moderating effects.

## 6 Conclusion

This study identified distinct family typologies among adolescents and demonstrated that the association between mobile phone addiction and depressive symptoms varies across family contexts. The findings highlight the importance of adopting a family-centered perspective in digital mental health research and intervention. In particular, adolescents from socioeconomically disadvantaged and structurally fragmented families may be especially vulnerable to the negative psychological effects of mobile phone addiction. These results suggest that interventions aimed at promoting adolescent digital well-being should extend beyond restricting phone use and instead strengthen family functioning and supportive resources. Furthermore, the family typologies identified through latent class analysis may provide a practical framework for identifying high-risk subgroups and guiding targeted prevention and intervention strategies.

## Data Availability

Publicly available datasets were analyzed in this study. This data can be found here: the data that support the findings of this study are not openly available due to reasons of sensitivity and are available from the corresponding author upon reasonable request.
