# Correction: Mobile phone addiction and depression among adolescents: the moderation effect of family environment

**DOI:** 10.3389/fpubh.2026.1925347

**Published:** 2026-07-22

**Authors:** Yanlin Bai, Xia Xu, Weijie Jiang, Yuting Yang, Qixi Li, Xiaoli Li, Qian Zhu, Yajing Meng, Changjian Qiu

**Affiliations:** 1 Mental Health Center, National Center for Mental Disorders, West China Hospital, Sichuan University, Chengdu, China; 2 Leshan Psychosomatic Hospital, Leshan, China; 3 Department of Sociology and Psychology, School of Public Administration, Sichuan University, Chengdu, China

**Keywords:** adolescent mental health, depression, family environment typology, latent class analysis, Mobile phone addiction, moderation effect

Authors Yanlin Bai and Xia Xu were erroneously omitted as equal contributing authors. “These authors have contributed equally to this work and share first authorship” has been added for these authors.

The original version of this article has been updated.

