# Supplementary material for: Mobile phone addiction and depression among adolescents: the moderation effect of family environment
Source: Front Public Health. 2026 Jun 30;14:1863623. doi: 10.3389/fpubh.2026.1863623 (PMC13364585; doi:10.3389/fpubh.2026.1863623)
Supplement: Supplementary file 1 [file Table_1.DOCX]

Supplementary Material

**Table S1 The Fitting of the latent class analysis model with different numbers of clusters**

| Numbers of clusters | AIC | BIC | CAIC | SABIC | AvePP | Entropy | Maximum log-likelihood | Predicted probability of membership | class-specific average posterior probabilities |
| --- | --- | --- | --- | --- | --- | --- | --- | --- | --- |
| 2 | 1302564 | 1302898 | 1302933 | 1302787 | 0.8813144 | 0.6085468 | -651246.9 | 0.4470416 0.5529584 | 0.8707 0.8899 |
| 3 | 1282240 | 1282747 | 1282800 | 1282578 | 0.8435214 | 0.6507778 | -641067.2 | 0.4067909 0.3526099 0.2405992 | 0.8731 0.8461 0.8076 |
| 4 | 1275975 | 1276653 | 1276724 | 1276427 | 0.789 | 0.6294729 | -637916.5 | 0.2635886 0.3907619 0.2186688 0.1269808 | 0.7676 0.8277 0.7708 0.8174 |
| 5 | 1271285 | 1272135 | 1272224 | 1271852 | 0.755 | 0.6122964 | -635553.4 | 0.32987081 0.15544795 0.21083235 0.20462291 0.09922599 | 0.7214 0.7285 0.7628 0.8102 0.7666 |
| 6 | 1269299 | 1270321 | 1270428 | 1269981 | 0.723 | 0.6110459 | -634542.7 | 0.24390126 0.17436510 0.12255232 0.09236190 0.08861698 0.27820244 | 0.7448 0.7292 0.7422 0.5727 0.7324 0.7399 |

AIC, Akaike information criterion. BIC, Bayesian information criterion. CAIC, consistent Akaike information criterion. SABIC, sample-size adjusted Bayesian

information criterion. AvePP, average latent class posterior probability.

**Table S2. Classification probability matrix for the three-class solution**

| Latent class (true) | Predicted class 1 | Predicted class 2 | Predicted class 3 |
| --- | --- | --- | --- |
| Latent_Class1 | 0.873120 | 0.043140 | 0.083740 |
| Latent_Class2 | 0.064776 | 0.846113 | 0.089111 |
| Latent_Class3 | 0.110011 | 0.082382 | 0.807607 |

**Table S3 Pairwise comparisons between the three latent classes**

|  | level | Overall | latent class 1 | latent class 2 | latent class 3 |  |  | 1 vs 2 |  | 1 vs 3 |  | 2 vs 3 |  |
| --- | --- | --- | --- | --- | --- | --- | --- | --- | --- | --- | --- | --- | --- |
|  |  | n=103874 | n=42255 | n=36627 | n=24992 | t/χ2 | p | t/χ2/z | p | t/χ2/z | p | t/χ2/z | p |
| gender (%) | F | 52326 (50.4) | 20142 ( 47.7) | 19196 ( 52.4) | 12988 ( 52.0) | 209.92 | <0.001 | 116.05 | <0.001 | 176.26 | <0.001 | 1.14 | 0.86 |
|  | M | 51548 (49.6) | 22113 ( 52.3) | 17431 ( 47.6) | 12004 ( 48.0) |  |  |  |  |  |  |  |  |
| age (mean (SD)) |  | 15.58 (1.74) | 15.38 (1.69) | 15.72 (1.78) | 15.73 (1.72) | 505.92 | <0.001 | 28.42 | <0.001 | 25.43 | <0.001 | 1.07 | 0.83 |
| PHQ-9.total.score (mean (SD)) |  | 6.36 (5.30) | 5.65 (4.94) | 6.07 (5.22) | 7.97 (5.67) | 1626.85 | <0.001 | 11.43 | <0.001 | 55.14 | <0.001 | 44.02 | <0.001 |
| MPAI.total.score (mean (SD)) |  | 31.49 (12.50) | 30.43 (11.98) | 30.51 (12.01) | 34.73 (13.49) | 1129.61 | <0.001 | 1.00 | 0.96 | 43.51 | <0.001 | 41.78 | <0.001 |
| inability.to.control.craving (mean (SD)) |  | 13.71 (5.78) | 13.22 (5.56) | 13.32 (5.58) | 15.12 (6.17) | 1008.12 | <0.001 | 2.49 | 0.04 | 41.50 | <0.001 | 38.45 | <0.001 |
| Withdrawal.and.escape (mean (SD)) |  | 6.26 (3.14) | 6.04 (2.97) | 6.06 (2.97) | 6.90 (3.53) | 712.42 | <0.001 | 0.86 | 1.00 | 34.48 | <0.001 | 32.42 | <0.001 |
| Anxiety.and.feeling.lost (mean (SD)) |  | 6.38 (3.16) | 6.24 (3.11) | 6.13 (3.02) | 6.98 (3.35) | 614.37 | <0.001 | 5.00 | <0.001 | 29.60 | <0.001 | 32.69 | <0.001 |
| Productivity.loss (mean (SD)) |  | 5.15 (2.65) | 4.93 (2.52) | 5.01 (2.54) | 5.73 (2.93) | 802.79 | <0.001 | 4.00 | <0.001 | 37.90 | <0.001 | 32.77 | <0.001 |
| Only.child(%) | no | 66365 (63.9) | 21010 ( 49.7) | 29718 ( 81.1) | 15637 ( 62.6) | 8417.80 | <0.001 | 1044.53 | <0.001 | 8434.74 | <0.001 | 2635.58 | <0.001 |
|  | yes | 37509 (36.1) | 21245 ( 50.3) | 6909 ( 18.9) | 9355 ( 37.4) |  |  |  |  |  |  |  |  |
| living.condition (%) | city | 48241 (46.4) | 33071 ( 78.3) | 6307 ( 17.2) | 8863 ( 35.5) | 33346.00 | <0.001 | 179.00 | <0.001 | 105.00 | <0.001 | 53.50 | <0.001 |
|  | country | 29895 (28.8) | 2397 ( 5.7) | 19266 ( 52.6) | 8232 ( 32.9) |  |  |  |  |  |  |  |  |
|  | villages | 25738 (24.8) | 6787 ( 16.1) | 11054 ( 30.2) | 7897 ( 31.6) |  |  |  |  |  |  |  |  |
| Cohabiting.relatives (%) | Both parents work outside the home (more than 6 months) and live with other relatives | 9787 ( 9.4) | 858 ( 2.0) | 1034 ( 2.8) | 7895 ( 31.6) | 54063.00 | <0.001 | 11.50 | <0.001 | 216.00 | <0.001 | 200.00 | <0.001 |
|  | live with parents | 71135 (68.5) | 37612 ( 89.0) | 31286 ( 85.4) | 2237 ( 9.0) |  |  |  |  |  |  |  |  |
|  | Living in a social welfare institution or other | 964 ( 0.9) | 106 ( 0.3) | 456 ( 1.2) | 402 ( 1.6) |  |  |  |  |  |  |  |  |
|  | One of the parents works outside the home (more than 6 months) and lives with the other parent | 21988 (21.2) | 3679 ( 8.7) | 3851 ( 10.5) | 14458 ( 57.9) |  |  |  |  |  |  |  |  |
| separation.more.than.6.months (%) | no | 65482 (63.0) | 35546 ( 84.1) | 29223 ( 79.8) | 713 ( 2.9) |  |  |  |  |  |  |  |  |
|  | yes | 38392 (37.0) | 6709 ( 15.9) | 7404 ( 20.2) | 24279 ( 97.1) |  |  |  |  |  |  |  |  |
| family.s.financial.situation (%) | general | 54410 (52.4) | 13993 ( 33.1) | 25079 ( 68.5) | 15338 ( 61.4) | 34315.00 | <0.001 | 178.00 | <0.001 | 121.00 | <0.001 | 37.50 | <0.001 |
|  | medium | 35257 (33.9) | 26764 ( 63.3) | 3076 ( 8.4) | 5417 ( 21.7) |  |  |  |  |  |  |  |  |
|  | poor | 12731 (12.3) | 102 ( 0.2) | 8472 ( 23.1) | 4157 ( 16.6) |  |  |  |  |  |  |  |  |
|  | rich | 1476 ( 1.4) | 1396 ( 3.3) | 0 ( 0.0) | 80 ( 0.3) |  |  |  |  |  |  |  |  |
| Gross.annual.household.income (%) | 150000-300000 | 11992 (11.5) | 10289 ( 24.3) | 0 ( 0.0) | 1703 ( 6.8) | 41420.00 | <0.001 | 58.90 | <0.001 | 44.70 | <0.001 | 7.76 | <0.001 |
|  | 60000-150000 | 40700 (39.2) | 23669 ( 56.0) | 6790 ( 18.5) | 10241 ( 41.0) |  |  |  |  |  |  |  |  |
|  | less than 60,000 | 47694 (45.9) | 5130 ( 12.1) | 29832 ( 81.4) | 12732 ( 50.9) |  |  |  |  |  |  |  |  |
|  | More than 300,000 | 3488 ( 3.4) | 3167 ( 7.5) | 5 ( 0.0) | 316 ( 1.3) |  |  |  |  |  |  |  |  |
| Satisfaction.with.family.relationships (%) | dissatisfied | 2814 ( 2.7) | 652 ( 1.5) | 694 ( 1.9) | 1468 ( 5.9) | 7152.60 | <0.001 | 15.70 | <0.001 | 82.10 | <0.001 | 66.20 | <0.001 |
|  | medium | 17161 (16.5) | 4557 ( 10.8) | 5691 ( 15.5) | 6913 ( 27.7) |  |  |  |  |  |  |  |  |
|  | satisfied | 32242 (31.0) | 12697 ( 30.0) | 10645 ( 29.1) | 8900 ( 35.6) |  |  |  |  |  |  |  |  |
|  | very dissatisfied | 1015 ( 1.0) | 219 ( 0.5) | 235 ( 0.6) | 561 ( 2.2) |  |  |  |  |  |  |  |  |
|  | very satisfied | 50642 (48.8) | 24130 ( 57.1) | 19362 ( 52.9) | 7150 ( 28.6) |  |  |  |  |  |  |  |  |

Note: latent class 1 = Participants in Middle-Class Stable Family; latent class 2 = Participants in Low-Income Stable Family; latent class 3 = Participants in Low-income vulnerable Family.

**Table S4 Gender differences within the latent class**

| LatentClass | Variable | Male | Female | t | p |
| --- | --- | --- | --- | --- | --- |
| 1 | PHQ-9.total.score(mean (SD)) | 5.00 (4.63) | 6.37 (5.17) | -28.57 | <0.001 |
|  | MPAI.total.score(mean (SD)) | 29.16 (11.21) | 31.81 (12.62) | -22.72 | <0.001 |
|  | inability.to.control.craving(mean (SD)) | 12.88 (5.36) | 13.58 (5.75) | -12.85 | <0.001 |
|  | Withdrawal.and.escape(mean (SD)) | 5.76 (2.78) | 6.36 (3.14) | -20.67 | <0.001 |
|  | Anxiety.and.feeling.lost(mean (SD)) | 5.84 (2.96) | 6.67 (3.22) | -27.24 | <0.001 |
|  | Productivity.loss(mean (SD)) | 4.68 (2.35) | 5.21 (2.67) | -21.64 | <0.001 |
| 2 | PHQ-9.total.score(mean (SD)) | 5.25 (4.88) | 6.82 (5.41) | -29.07 | <0.001 |
|  | MPAI.total.score(mean (SD)) | 29.20 (11.38) | 31.70 (12.44) | -20.11 | <0.001 |
|  | inability.to.control.craving(mean (SD)) | 12.96 (5.44) | 13.64 (5.69) | -11.79 | <0.001 |
|  | Withdrawal.and.escape(mean (SD)) | 5.79 (2.80) | 6.30 (3.09) | -16.60 | <0.001 |
|  | Anxiety.and.feeling.lost(mean (SD)) | 5.70 (2.86) | 6.51 (3.12) | -25.93 | <0.001 |
|  | Productivity.loss(mean (SD)) | 4.75 (2.39) | 5.24 (2.64) | -18.82 | <0.001 |
| 3 | PHQ-9.total.score(mean (SD)) | 7.05 (5.39) | 8.82 (5.79) | -25.06 | <0.001 |
|  | MPAI.total.score(mean (SD)) | 33.14 (12.87) | 36.20 (13.89) | -18.09 | <0.001 |
|  | inability.to.control.craving(mean (SD)) | 14.70 (5.99) | 15.52 (6.30) | -10.53 | <0.001 |
|  | Withdrawal.and.escape(mean (SD)) | 6.55 (3.36) | 7.23 (3.65) | -15.38 | <0.001 |
|  | Anxiety.and.feeling.lost(mean (SD)) | 6.48 (3.21) | 7.43 (3.41) | -22.71 | <0.001 |
|  | Productivity.loss(mean (SD)) | 5.41 (2.80) | 6.02 (3.01) | -16.60 | <0.001 |

Note: latent class 1 = Participants in High-Resource Stable Family, latent class 2 = Participants in Low-Resource Cohesive Family, latent class 3 = Participants in Low-Resource Fragmented Family.

P value: Comparison among 3 groups

**Table S5.Simple slope analyses by latent class**

|  | latent class | Simple Slope | 95%LCI | 95%UCI | SE | P |
| --- | --- | --- | --- | --- | --- | --- |
| total participants | 1 | 0.239126953 | 0.235807577 | 0.242446329 | 0.001693571 | <0.001 |
|  | 2 | 0.262553891 | 0.259019046 | 0.266088735 | 0.001803504 | <0.001 |
|  | 3 | 0.247660858 | 0.243870167 | 0.25145155 | 0.001934039 | <0.001 |
|  |  |  |  |  |  |  |
| junior male | 1 | 0.238541518 | 0.232431604 | 0.244651431 | 0.003117238 | <0.001 |
|  | 2 | 0.246319636 | 0.239527971 | 0.2531113 | 0.003465063 | <0.001 |
|  | 3 | 0.251172921 | 0.244032252 | 0.258313589 | 0.003643122 | <0.001 |
|  |  |  |  |  |  |  |
| junior female | 1 | 0.277398475 | 0.271249937 | 0.283547012 | 0.003136941 | <0.001 |
|  | 2 | 0.304320003 | 0.297968661 | 0.310671346 | 0.003240411 | <0.001 |
|  | 3 | 0.286623301 | 0.279671649 | 0.293574953 | 0.003546685 | <0.001 |
|  |  |  |  |  |  |  |
| senior male | 1 | 0.221222297 | 0.213489338 | 0.228955255 | 0.003945229 | <0.001 |
|  | 2 | 0.240245053 | 0.231807951 | 0.248682155 | 0.004304471 | <0.001 |
|  | 3 | 0.221765694 | 0.212789481 | 0.230741908 | 0.004579517 | <0.001 |
|  |  |  |  |  |  |  |
| senior female | 1 | 0.21491848 | 0.207345979 | 0.222490981 | 0.003863379 | <0.001 |
|  | 2 | 0.228691223 | 0.220947104 | 0.236435341 | 0.003950936 | <0.001 |
|  | 3 | 0.215794849 | 0.207677981 | 0.223911718 | 0.004141108 | <0.001 |

SE,Standard Error. CI, Confidence Interval.

**Table S6. Predicted outcome scores across latent classes**

|  | contrast | estimate | SE | t | P |
| --- | --- | --- | --- | --- | --- |
| total participants | Latent Class 1（w1） - Latent Class 2（w2） | -0.331052 | 0.029567 | -11.196501 | <0.001 |
|  | Latent Class 1（w1） - Latent Class 3（w3） | -1.165609 | 0.033483 | -34.812392 | <0.001 |
|  | Latent Class 2（w2） - Latent Class 3（w3） | -0.834556 | 0.034333 | -24.307927 | <0.001 |
|  |  |  |  |  |  |
| junior male | Latent Class 1（w1） - Latent Class 2（w2） | -0.051562 | 0.050135 | -1.028454 | <0.001 |
|  | Latent Class 1（w1） - Latent Class 3（w3） | -0.973122 | 0.058502 | -16.633907 | <0.001 |
|  | Latent Class 2（w2） - Latent Class 3（w3） | -0.921560 | 0.061294 | -15.035075 | <0.001 |
|  |  |  |  |  |  |
| junior female | Latent Class 1（w1） - Latent Class 2（w2） | -0.253229 | 0.055211 | -4.586550 | <0.001 |
|  | Latent Class 1（w1） - Latent Class 3（w3） | -1.221559 | 0.064460 | -18.950518 | <0.001 |
|  | Latent Class 2（w2） - Latent Class 3（w3） | -0.968330 | 0.065778 | -14.721270 | <0.001 |
|  |  |  |  |  |  |
| senior male | Latent Class 1（w1） - Latent Class 2（w2） | -0.534507 | 0.067499 | -7.918721 | <0.001 |
|  | Latent Class 1（w1） - Latent Class 3（w3） | -1.189752 | 0.073933 | -16.092399 | <0.001 |
|  | Latent Class 2（w2） - Latent Class 3（w3） | -0.655244 | 0.076824 | -8.529131 | <0.001 |
|  |  |  |  |  |  |
| senior female | Latent Class 1（w1） - Latent Class 2（w2） | -0.789640 | 0.067135 | -11.762048 | <0.001 |
|  | Latent Class 1（w1） - Latent Class 3（w3） | -1.390097 | 0.073032 | -19.034199 | <0.001 |
|  | Latent Class 2（w2） - Latent Class 3（w3） | -0.600457 | 0.072462 | -8.286533 | <0.001 |
